# Supplementary material for: The Construction and Exploration of a Comprehensive MicroRNA Centered Regulatory Network in Foxtail Millet (Setaria italica L.)
Source: Front Plant Sci. 2022 May 6;13:848474. doi: 10.3389/fpls.2022.848474 (PMC9121102; doi:10.3389/fpls.2022.848474)
Supplement: Supplementary file 3 [file Data_Sheet_3.zip › Sit-miR171f_Seita.9G097600.1_18_TPlot.pdf]

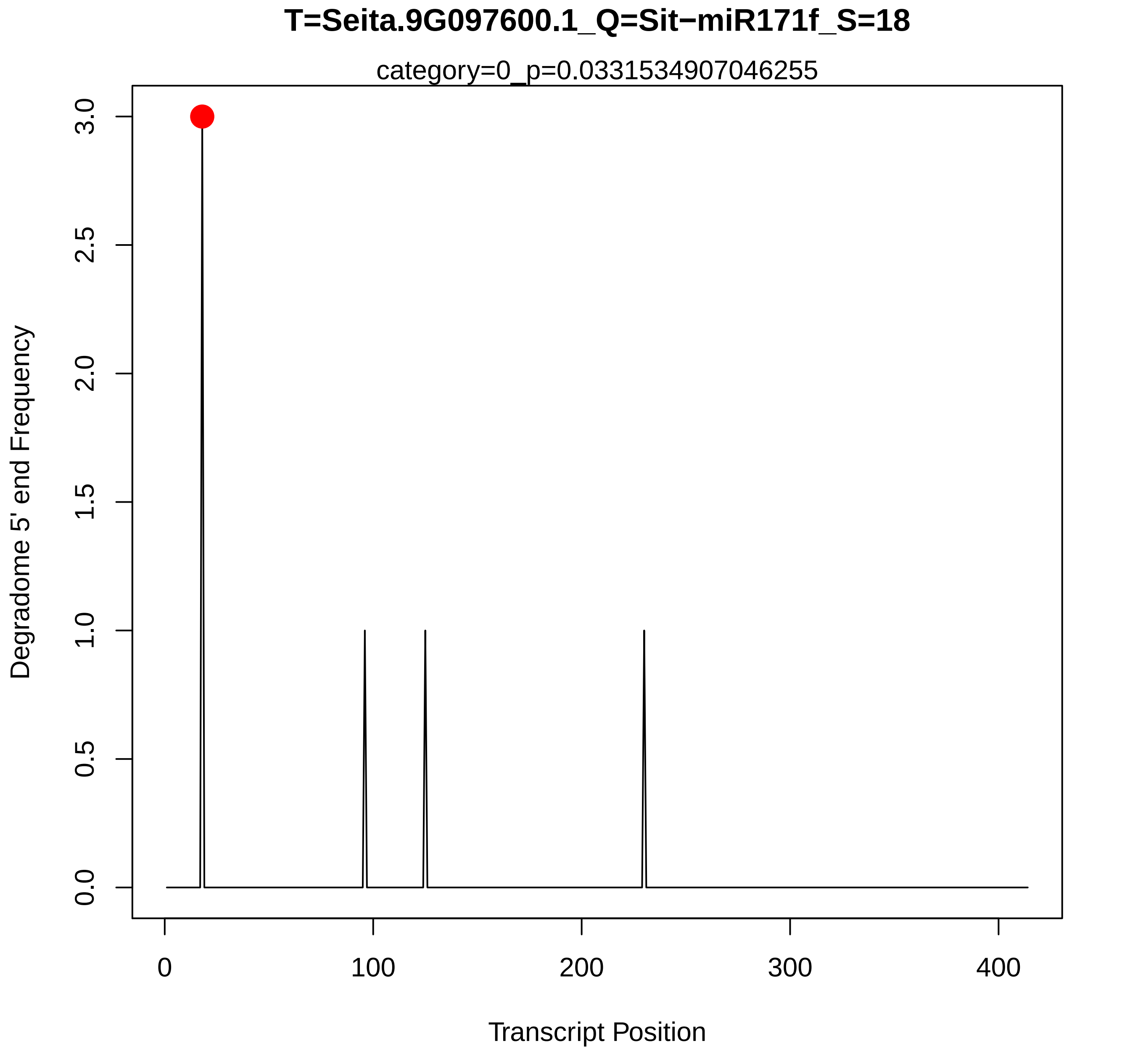
T=Seita.9G097600.1_Q=Sit−miR171f_S=18
category=0_p=0.0331534907046255
Degradome 5' end Frequency
0.0
0.5
1.0
1.5
2.0
2.5
3.0
0
100
200
300
400
Transcript Position
